# Enhanced washing of polycyclic aromatic hydrocarbons from contaminated soils by the empowered surfactant properties of de novo O-alkylated humic matter

**DOI:** 10.1007/s11356-024-32292-3

**Published:** 2024-02-08

**Authors:** Alessandro Piccolo, Marios Drosos, Assunta Nuzzo, Vincenza Cozzolino, Antonio Scopa

**Affiliations:** 1 Department of Agricultural Sciences, University of Naples Federico II, Via Università 100, 80055 Portici, Italy; 2 School of Agricultural, Forestal, Food and Environmental Sciences (SAFE), University of Basilicata, Viale Dell’Ateneo Lucano 10, 85100 Potenza, Italy

**Keywords:** Humic acid, Polycyclic aromatic hydrocarbon, Phase transfer alkylation, Soil washing, Synthetic surfactants, Consecutive washings

## Abstract

Aqueous solutions of humic acid (HA) derivatized by a catalyzed O-alkylation reaction with methyl, pentyl, and benzyl groups at 40, 60, and 80% of total HA acidity were used to wash off polycyclic aromatic hydrocarbons (PAHs) from two contaminated soils. The enhanced surfactant properties enabled the alkylated HA to remove phenanthrene, anthracene, fluoranthene, and pyrene from both soils more extensively than the original unmodified HA, the 60% benzylation generally showing the greatest soil washing efficiency. For both soils, all alkylated HA revealed greater PAH removals than Triton X-100 nonionic surfactant, while the benzylated and methylated HA nearly and fully matched pollutants release by the anionic SDS in the coarse- and fine-textured soils, respectively. A consecutive second washing with 60% benzylated HA removed additional PAHs, in respect to the first washing, from the coarser-textured soil, except for fluoranthene, while removal from the finer-textured soil incremented even more for all PAHs. These findings indicate that the enhanced hydrophobicity obtained by a simple and unexpensive chemical derivatization of a natural humic surfactant can be usefully exploited in the washing of polluted soils, without being toxic to the soil biota and by potentially promoting the subsequent bio-attenuation of organic pollutants.

**Supplementary Information:**

The online version contains supplementary material available at 10.1007/s11356-024-32292-3.

## Introduction

Polycyclic aromatic hydrocarbons (PAHs) are major contaminants of polluted soils and represent a considerable health and environmental hazard due not only to their carcinogenic, mutagenic, and teratogenic properties but also to their great persistence in soils conferred by a high hydrophobicity (Lau et al. 2014). While PAHs are largely produced during incomplete combustion of hydrocarbon-containing fuels, primary anthropogenic sources are open fires, domestic heating systems, and manufactured gas plants (Sakshi Singh and Haritash 2019). In the latter sites, PAH concentration in soil may be significant and ranging from 724 to 7700 ppm (Gong et al. 2006).

Since toxicity and hydrophobicity of PAHs show a parallel increase with the number of rings, a strategy for detoxification of contaminated soils should address the alteration of the hydrophobic interactions which stabilize adsorption of such highly hydrophobic pollutants on surfaces of soil particles. Several techniques were developed based on this principle, such as electrokinetic remediation, solvent extraction, and washing with surfactants (Vidal and Báez 2023). In the case of soil washing, both anionic and nonionic synthetic surfactants with amphiphilic properties are used as additives to water to enhance the extraction efficiency of PAHs from soil (dos Santos et al. 2023). However, the use of synthetic surfactants to remediate contaminated sites is limited by their intrinsic toxicity towards soil microorganisms that hinders further biodegradation of pollutants and a balanced biological activity in the washed soils (Deschênes et al. 1995; Sandbacka et al. 2000).

In alternative, the use of biosurfactants in washings of polluted soils have been increasingly proposed in recent years (Bezza and Chirwa 2017; Vijayakuma and Saravanan 2015), offering the advantage over chemical surfactants to be easily biodegradable and biologically safe (Mulligan 2005). Moreover, biosurfactants may enhance the bioavailability of the residual molecules remaining sorbed on soil particles after washing (Whang et al. 2008). The combination of soil washing followed by biodegradation induced by biosurfactants may thus represent an effective means of soil remediation from PAH pollution (Chebbi et al. 2017; Lamichhane et al. 2017). Among biosurfactants, humic substances (HS) of different origin have been recognized to be capable of incorporating PAHs (Conte et al. 2001), increasing aerobic degradation of recalcitrant soil contaminants (Berselli et al. 2004; Fava and Piccolo 2002; Fava et al. 2004) and effectively washing away pollutants from soils (Conte et al. 2005; Piccolo et al. 2019a, 2021; Wei et al. 2023).

End products of the biotic degradation of animal and vegetal biomasses, HS are supramolecular associations of a multitude of heterogeneous molecules of relatively small mass held together by weak interactions such as dispersive forces and hydrogen bonds (Nebbioso and Piccolo 2011; Piccolo 2002; Piccolo et al. 2019b; Wells 2019). The self-assembling of hydrophilic and hydrophobic molecules into pseudo-micellar superstructures confers to HS the surfactant properties that enable the thermodynamically favorable repartition of PAHs from soil adsorption sites into the humic hydrophobic domains and efficiently wash such apolar pollutants from soils (Balasubramanian et al. 1989; Smejkalova and Piccolo 2008; Tschapek et al. 1981).

However, the capacity of HS as biosurfactants in cleaning-up contaminated sites may depend on soil texture. In fact, soils rich in fine-sized particles (silt and clay) may reduce substantially the washing efficiency of surfactants (Kuhlman and Greenfield 1999; Lee et al. 2002). This limitation may be overcome by increasing the hydrophobicity of HS through a chemical modification of humic molecules. Different derivatization reactions of HS have been previously performed with various aims: (i) solubility increase in water (Terashima et al. 2007); (ii) soil aggregates stabilization (Kulikova et al. 2021); (iii) redox properties change (Volikov et al. 2021); (iv) alteration of conformational structure (Nebbioso and Piccolo 2015). Recently, a phase-transfer catalyzed O-alkylation reaction was reported to vary the hydrophobicity of HS by covalently linking methyl, pentyl, and benzyl residues to the oxygen-containing humic functional groups (Piccolo et al. 2023).

In this study, we employed O-alkylated HS as soil washing biosurfactants on two soils of different texture, which were spiked with four PAHs of varying polycondensation, such as anthracene, phenanthrene, fluoranthene, and pyrene. The aim was to verify whether the enhanced affinity of the chemically modified humic matter towards hydrophobic PAHs improved the washing of the two soils in comparison to the original HS and two different synthetic surfactants.

## Materials and methods

### Soils and humic matter

Soils were collected from the surface layers (0–20 cm) of (1) a sandy-clay-loam Typic Haploxeralf (Soil 1) with 8.9% OC and 47.0, 20.1, and 32.9% of sand, silt, and clay, respectively, and (2) a clay-loam Vertic Xerofluvent with 1.05% OC and 36.6, 33.75, and 29.65% of sand, silt, and clay, respectively. Soils were sampled at the University of Napoli Federico II experimental stations of Torre Lama, near Salerno (Soil 1), and Castel Volturno near Caserta (Soil 2). Soil samples were air-dried and sieved at 2.00 mm before use.

The humic acid (HA) used in this study was extracted from a Leonardite source (TEMA, Tecnología Especializada en el Medio Ambiente, https://www.temamexico.mx/), and details on HA isolation and characterization are reported elsewhere (Piccolo et al. 2023). An automatic titrator (TIM840 Titration Manager, Radiometer Analytical, France) was used to measure total acidity. A suspension of 0.5 mg mL^−1^ of HA in deionized water was titrated to pH 9.0 with 0.5 M NaOH under a N_2_ stream under stirring. The calculated HA total acidity was 6.05 meq H^+^ g^−1^.

### Phase-transfer O-alkylation reaction

Details on the derivatization reaction to modify the hydrophobicity of humic materials were previously reported (Piccolo et al. 2023). Briefly, HA were dissolved in deionized water, the pH was adjusted to 9.0 with a 1.5 M NaOH solution, and the phase-transfer catalyst TBAH (tetrabutylammonium hydroxide) (Bu_4_N^+^OH^−^) was added to the solution. After 2 h stirring at room temperature, specific volumes of each alkyl halide (methyl iodide; pentyl bromide; benzyl bromide) were added to the humic solution in amounts corresponding to 40, 60, and 80% of HA total acidity, in order to partially and progressively saturate the nucleophilic humic sites, and concomitantly maintain the aqueous solubility of the modified humic matter. The reaction mixture was stirred for 2 h at room temperature. Then, the pH was adjusted to 1.0 with a 10% HCl solution to precipitate the reaction products. The excessive alkylating agent was removed under reduced pressure at 50–70 °C, while the residual tetrabutylammonium salts were removed from the reaction products by washing the residue with hot (45 °C) deionized water. Finally, the residue was dialyzed against deionized water and freeze dried. All reagents 98–99% pure were purchased from Aldrich (Milano, Italy) and used without further purification.

### Soil spiking

Soils were spiked according to the method reported by Sawada et al. (2004) with each selected polycyclic aromatic hydrocarbon (PAH): phenanthrene (PHE), anthracene (ANT), fluoranthene (FLA), and pyrene (PYR). Briefly, 250 g of dry soil and 1 L of acetone containing 2500 mg L^−1^ of PHE, ANT, and PYR and 1250 mg L^−1^ of FLA were mixed in a 2000-mL round-bottomed flask and shaken for 4 h in a rotary shaker at 50 rpm. The acetone was evaporated with a rotary evaporator at 30–35 °C, and the soils were then air-dried for 2 days under dark conditions in a fume hood. The content of PHE, ANT, FLA, and PYR was revealed to be, respectively, 9.1, 10.0, 4.8, and 9.9 g kg^−1^ for the dry soil 1, and 9.8, 10.2, 5.1, and 10.6 g kg^−1^ for the dry soil 2, with a standard deviation never exceeding ± 0.2.

### Soil washing

Each soil was subjected to soil washing by a water solution (control), a solution of original unmodified HA, and solutions of methylated, pentylated, and benzylated HA at 40, 60, or 80% of humic acid total acidity. The solution concentration of original and modified HA was 4 g L^−1^. Other washings of spiked soils were also conducted with synthetic surfactants, such as 4% (w/v) of the anionic sodium dodecyl sulfate (SDS), and 4% (v/v) of the nonionic polyethylene glycol *tert*-octylphenyl ether (Triton X-100) solutions. Triplicates of both soils spiked with PAHs (10 g each) were placed in Erlenmeyer flasks and suspended in 200 mL of different soil washing solutions and shaken for 24 h in a rotary shaker at 50 rpm. All suspensions were centrifuged in Teflon tubes at 10,000 rpm for 10 min to separate soil residues from washing solutions. The soil residues were oven dried at 35 °C and stored in a desiccator before further treatments.

Triplicates (10 g) of both soils underwent two consecutive washings with the aqueous solution of benzylated HA at 60% of total acidity by first washing with the benzylated HA solution (4 g L^−1^) as by the above procedure. The resulting dried residue was again subjected to a second washing with the 60% benzylated HA solution. This second residue of both soils was again oven-dried and stored in a desiccator for further analyses.

### Ultrasonic extraction

Ultrasonication had been previously proved as the most efficient method to solvate organic pollutants from contaminated soils (Conte et al. 2005). Both unwashed and washed spiked soils (10 g) were suspended in 100 mL of an acetone/dichloromethane (1:1) mixture and sonicated with a Misonix XL2020 sonicator, as by the procedure outlined in USEPA Test Method 3550 B. A power of 55 W was applied for 12 min to the soil suspensions to obtain a total energy of 39.6 kJ. After sonication, the suspension was centrifuged in Teflon tubes at 10,000 rpm for 10 min to separate the soil residue from the supernatant, whose PAH content was analyzed by GC–MS. The removed percentage of PAHs was calculated based on the content of initial PAHs spiked on soils and final content left in soils after the washings.

### GC–MS analysis

A Perkinelmer autosystem™ XL gas-chromatograph, equipped with a Programmed-Temperature Split/Splitless injector with programmable pneumatic control kept at a constant temperature of 250 °C, a 30-m-long, 0.25-mm ID, Restek Rtxc-5MS capillary column (5% diphenyl-95% dimethylpolysiloxane), and a Perkin-Elmer TurboMass Gold mass-spectrometer were used for qualitative and quantitative analysis of contaminants in the soil extracts. The conditions used for GC analyses were the following: (1) initial temperature of 40 °C for 4 min; (2) to 270 °C at a 10 °C/min rate; (3) isothermal for 3 min. The total GC run time was 30 min. Helium was the carrier gas at 1.5 mL min^−1^ with a split-flow of 30 mL min^−1^. The inlet-line temperature of the GC–MS system was set at 250 °C, while that of the MS source at 300 °C. A solvent delay time of 5 min was applied before acquisition of the mass spectra to prevent filament injuries. Low and high m/z limits of the mass spectrometer were 50, and 400 µm, respectively. A NIST mass spectral library version 1.7 was used for peak identification.

### Data treatment and statistical analysis

Sonication extractions were performed in triplicate for each soil before and after soil washings. Each organic extract was analyzed in triplicate by GC–MS analysis. Quantitative results by GC–MS analyses were weight-averaged to provide experimental error. The least significant difference (LSD) test was used to determine the statistical significance of PAH removal. Data were significantly different among values if *p*(*F*) < 0.05. Version 8.0 of Design expert was used for all statistical analyses, including multiple comparisons test of Duncan, Fisher (LSD) and Tukey–Kramer (*P* < 0.05).

## Results and discussion

### Soil washing with HA and alkylated HA

The efficiency in the soil washing treatments by water and aqueous solutions of either unmodified or various alkylated HA was evaluated as percent removal of the four different PAHs (PHE, ANT, FLA, and PYR) from the two soils of this study (Table 1). The aqueous solution of the original unmodified HA washed more PAHs than just water, being the removal the largest for ANT and the least for FLA in both soils (Table 1). Under this treatment, the sandy-clay-loam soil 1 generally released more pollutants than the clay-loam soil 2, except for FLA that was hardly removed from both soils.

**Table 1:** Percent of PAH removal (± SD) from soil 1 and soil 2 by washings with water, aqueous solutions of unmodified original HA, and aqueous solutions of alkylated HA at 40, 60, and 80% of total acidity

	Phenanthrene	Anthracene	Fluoranthene	Pyrene
	Soil 1			
	Methylated HA			
Water	16.0 ± 1.8	17.2 ± 0.3	0.6 ± 0.6	0.5 ± 0.6
Unmodified HA	26.1 ± 0.1	43.1 ± 0.8	0.5 ± 0.3	16.8 ± 2.2
40	41.2 ± 2.2	56.7 ± 0.6	9.1 ± 2.0	39.5 ± 2.0
60	42.6 ± 0.3	54.6 ± 0.2	8.8 ± 2.3	40.6 ± 0.1
80	35.0 ± 2.6	54.4 ± 1.4	6.3 ± 2.9	35.5 ± 2.8
	Pentylated HA			
Unmodified HA	26.1 ± 0.1	43.1 ± 0.8	0.5 ± 0.3	16.8 ± 2.2
40	44.6 ± 1.3	53.3 ± 0.1	8.9 ± 1.0	39.9 ± 1.3
60	42.6 ± 0.2	53.5 ± 0.9	13.1 ± 0.3	36.6 ± 0.4
80	41.2 ± 0.7	52.4 ± 0.1	4.8 ± 0.8	34.9 ± 0.9
	Benzylated HA			
Unmodified HA	26.1 ± 0.1	43.1 ± 0.8	0.5 ± 0.3	16.8 ± 2.2
40	39.4 ± 0.1	45.7 ± 0.3	9.4 ± 3.6	30.7 ± 1.5
60	49.8 ± 0.4	49.4 ± 1.1	19.9 ± 1.5	48.9 ± 0.1
80	45.4 ± 1.4	45.6 ± 1.0	13.0 ± 1.5	39.9 ± 0.7
	Soil 2			
	Methylated HA			
Water	18.7 ± 0.6	17.9 ± 2.4	0.5 ± 0.3	6.5 ± 0.8
Unmodified HA	18.0 ± 0.4	35.0 ± 1.6	0.6 ± 0.5	11.1 ± 0.2
40	39.3 ± 1.2	46.5 ± 0.1	3.7 ± 0.4	33.6 ± 0.8
60	42.9 ± 0.7	55.2 ± 0.5	10.7 ± 0.3	41.7 ± 0.4
80	42.6 ± 1.4	55.0 ± 1.1	10.3 ± 2.4	41.4 ± 0.8
	Pentylated HA			
Unmodified HA	18.0 ± 0.4	35.0 ± 1.6	0.6 ± 0.5	11.1 ± 0.2
40	32.3 ± 0.5	40.3 ± 3.1	0.2 ± 0.2	25.9 ± 2.1
60	35.0 ± 1.2	41.0 ± 0.7	1.9 ± 0.7	26.4 ± 0.1
80	31.7 ± 1.2	41.1 ± 0.9	2.1 ± 2.1	25.4 ± 1.3
	Benzylated HA			
Unmodified HA	18.0 ± 0.4	35.0 ± 1.6	0.6 ± 0.5	11.1 ± 0.2
40	30.6 ± 2.3	47.4 ± 0.6	0.4 ± 0.5	21.7 ± 2.6
60	36.9 ± 0.1	48.0 ± 1.6	8.9 ± 0.3	33.4 ± 0.5
80	30.7 ± 2.2	48.5 ± 0.3	3.0 ± 2.4	27.2 ± 1.5

The washing of the two soils with aqueous solutions of methylated HA resulted in much larger removal of PAHs than for the unmodified HA (Table 1; Fig. S1). Again, and regardless of the soil type, anthracene was the most removed pollutant, while fluoranthene was the least one. For soil 1, the modified HA enhanced the efficiency of PAHs removal when the methylation satisfied both 40 and 60% of the HA total acidity, but it was somewhat decreased at 80% of methylation (Table 1; Fig. S1). In particular, the percent increase of PAH removal at 40/60% of methylation over that of the original HA passed from 57.9/63.2, 31.6/26.7, 1720/1660, 135.1/141.7% to 34.1, 26.2, 1160, 111.3% at 80% of methylation for PHE, ANT, FLA, and PYR, respectively (Table S1). For soil 2, the washing of PAHs with HA methylated at 40% of total acidity was significantly less efficient than for 60 and 80% of methylation (Table 1; Fig. S1). In fact, the percent increase in PHE, ANT, FLA, and PYR removal at 40% of methylation in respect to that of the unmodified HA, resulted, respectively, 118.3, 32.9, 516.7, 202.7%, whereas that found for 60/80% of methylation raised to 138.3/136.7, 57.7/57.1, 1683.3/1616.7, and 275.7/273% (Table S1).

A similar trend of efficiency was observed when soils were washed with solutions of pentylated HA, although the percent removal of PAHs was more extensive for soil 1 than for soil 2 (Table 1; Fig. S3 and S4). For soil 1, the greatest removal of anthracene and pyrene, in respect to the original HA, occurred by washing with 40% of HA pentylation, whereas PHE and FLA were mostly released by the action of the pentylated HA at 60% of total acidity (Table 1 and S1; Fig. S3 and S4). In the case of soil 2, the soil washing with pentylated HA was generally less efficient than for soil 1 and the most extensive release of pollutants, as compared to the original HA, was generally obtained by the 60% pentylated HA, that succeeded to solvate 35, 41, 1.9, and 26.4% of PHE, ANT, FLA, and PYR, respectively (Table 1).

The covalent insertion of benzyl groups into the HA molecular system provided a similar soil washing efficiency as the methylated and pentylated HA, except for a generally greater FLA solubilization (Table 1; Fig. S5 and S6). For soil 1, the percent increase of pollutant extraction by washing with the 60% benzylated HA resulted generally the largest, in respect to either the original HA or both methylated and pentylated HA at the same percent of total acidity, except for ANT (Table S1). In particular, the affinity of the benzylated HA to FLA was the largest of all modified HA and capable to solvate the greatest amount of this pollutant at 60% of benzylation (Table 1; Fig. S5 and S6), with a percent increase as large as 3880%, in comparison to the unmodified HA (Table S1). For soil 2, benzylation of HA at 60% of total acidity washed a larger percentage of PAHs than for benzylated HA at 40 and 80% (Table 1; Fig. S5 and S6), although, in comparison to the original HA, its efficiency in extracting pollutants from this soil remained greater and smaller than for the pentylated and methylated HA, respectively, at the same percent of alkylation (Table S1).

Our findings indicate that the PAHs mobilized from soil by alkylated HA was either similar to previous soil washing results using different biosurfactants (Zhu et al. 2023) or even larger (Cazals et al. 2022). We also verified that the original unmodified HA washed less PAHs from the heavier texture soil 2 than the lighter textured soil 1, as expected from previous literature that reports a reduced efficiency of surfactants in soils with fine-sized particles (Lee et al. 2002). In these soils, the adsorption of hydrophobic organic matter on the surface of fine colloidal particles favors the transition from micro into macroaggregates (Jastrow 1996; Piccolo et al. 2019a), thereby protecting the co-adsorbed PAHs from being reached by the humic washing solutions and limiting the pollutants removal, that was even less than 1% in the case of the highly hydrophobic FLA (Table 1).

All alkylated derivatives at 40, 60, and 80% of HA total acidity increased removal of PAHs from soils in respect to the original HA, due to the greater hydrophobicity conferred to the humic surfactant by the insertion of alkyl groups (Piccolo et al. 2023). Such enhanced surfactant properties of modified HA may have induced a disruption of soil aggregates and favored the release of apolar PAHs (Smejkalova and Piccolo 2008). Nevertheless, the repartition of PAHs from soil surfaces into the hydrophobic domains of alkylated HA was still dependent on the soil texture and was less efficient for the finer-textured soil 2 than for the coarser soil 1.

The mechanism of increasing hydrophobicity appeared to be effective in solvating PAHs by HA derivatives at all rates of humic total acidity, although the most efficient pollutant removal from both soils, as compared to the unmodified HA, generally occurred at 60% alkylation (Table S1). At this rate, the proton displacement by either methyl, pentyl, or benzyl groups in the O-alkylation reaction of HA was found to be more extensive (Piccolo et al. 2023). This led to a more stable system, as it is proven by the general lesser standard deviation for the percent increase of PAH removal at 60% alkylation than for those at 40 and 80% (Table S1). In particular, the fact that the 60% benzylated HA promoted the largest removal of the highly recalcitrant fluoranthene from soil 1 and a significant release from soil 2 (Table 1) implies that the mechanism of hydrophobic repartition driven by weak dispersive forces is also accompanied, for the benzylated derivative, by that of multiple π-π bindings between the FLA aromatic rings and the benzyl groups introduced in HA. The counterintuitive fact that HA alkylated at 80% of total acidity was generally less efficient than 60% alkylation in removing pollutants from both soils should be attributed to the conformational dynamics of the supramolecular structure of humic matter that becomes tighter with increasing hydrophobicity, thereby reducing PAH repartition into the HA apolar domains and sites availability to π-π interactions.

### Original and alkylated HA versus synthetic surfactants

**Fig. 1:**
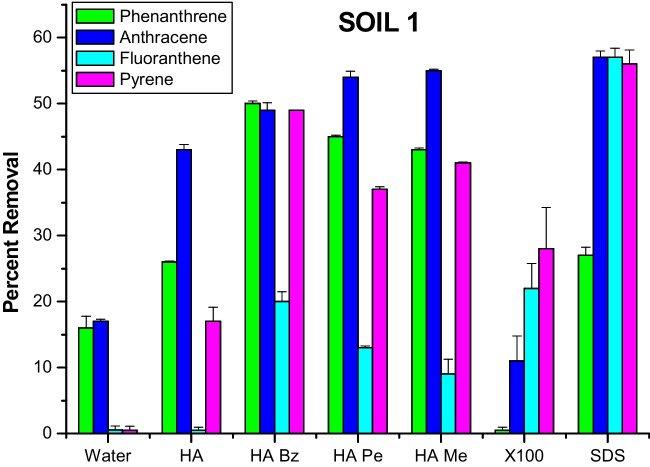
Percent removal of four PAHs from soil 1 by washing with water, aqueous solutions of unmodified original HA, of alkylated HA at 60% of total acidity and of X100 and SDS synthetic surfactants

**Fig. 2:**
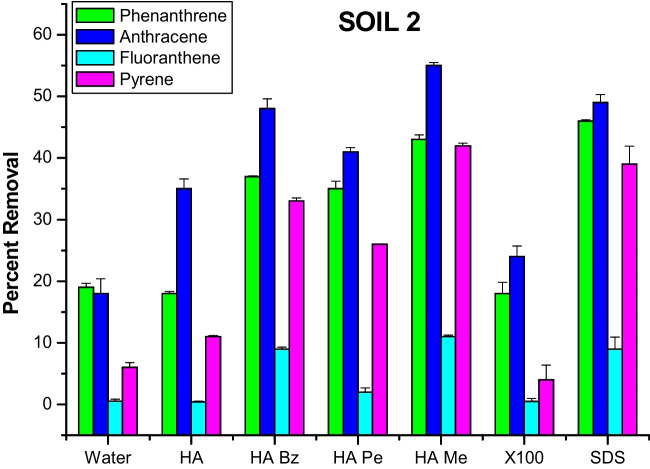
Percent removal of four PAHs from soil 2 by washing with water, aqueous solutions of unmodified original HA, of alkylated HA at 60% of total acidity and of X100 and SDS synthetic surfactants

Despite their deleterious effects on soil microbial bioactivity and biodiversity, synthetic surfactants are still widely used in remediation of soils contaminated by PAHs since, like biosurfactants, they remove organic pollutants by repartition into the surfactant micellar phases formed in water (Chun et al. 2002; Cuypers et al. 2002; Trellu et al. 2018; Chen et al. 2021). For the two soils of this study, we compared the washing efficiency of synthetic surfactants (TX-100 and SDS) with that observed for original HA and for the more performing alkylated derivatives at 60% of total acidity (Figs. 1 and 2).

Anthracene was removed in greater amount by the unmodified HA than by TX-100 in both soils, while phenanthrene was released more in soil 1 and to a similar extent in soil 2. Conversely, fluoranthene and pyrene were solvated more in TX-100 than in the original HA for soil 1, whereas for soil 2, the unmodified HA was more effective on pyrene than TX-100 and fluoranthene was not significantly different between the two (Figs. 1 and 2). Moreover, the SDS synthetic surfactant was capable to remove from both soils substantially more PAHs than the unmodified HA.

In respect to the original HA, the improvement in washing efficiency was dramatic for the alkylated HA, which showed for both soils a significant increase of PAH removal than for TX-100, except for fluoranthene in soil 1 that was solvated by the synthetic surfactant in similar amount as the benzylated HA (Figs. 1 and 2). In the case of SDS, its washing efficiency for soil 1 was invariable greater than all alkylated HA, among which the benzylated derivative was the most effective. For soil 2, SDS washed off more PHE than all alkylated HA, but the methylated HA removed more ANT and FLA than SDS, and both benzylated HA and SDS solvated equal amounts of FLA.

These results confirm that the modification of HA by O-alkylation reactions increases the heterogeneous hydrophobicity of humic micellar phases in aqueous solutions, thereby exerting a capacity to solvate from soil PAHs of different structures with an efficiency often similar to, if not as large as, the synthetic surfactants (Fava et al. 2004). However, it appears that repartition of PAHs into hydrophobic micellar domains was more effective for SDS than for Triton X-100, whose pollutant removing capacity was generally inferior to that of alkylated HA. This was particularly evident for methylated and benzylated HA which could account their washing effectiveness not only to the cited mechanism of hydrophobic drive of contaminants into flexible HA apolar domains but also to the additional mechanism of π-π interactions between aromatic rings in both PAHs and humic matter. It has been previously suggested that humic materials enriched with aromatic groups are the most efficient detoxifying agents in respect to PAHs (Perminova et al. 1999), since the latter bind humic substances by both specific affinity to aromatic moieties and nonspecific partitioning in hydrophobic domains (Kile and Chiou 1989; Chiou et al. 1998). The occurrence of both mechanisms was confirmed by experiments in which hydrophobic interactions were found responsible for the removal of pyrene from soils (Chefetz et al. 2000), and an induced bleaching of HA proved that aromatic structures could not solely contribute to the sorption of hydrophobic compounds to HA (Simpson et al. 2003).

### PAH removal by consecutive soil washings

**Fig. 3:**
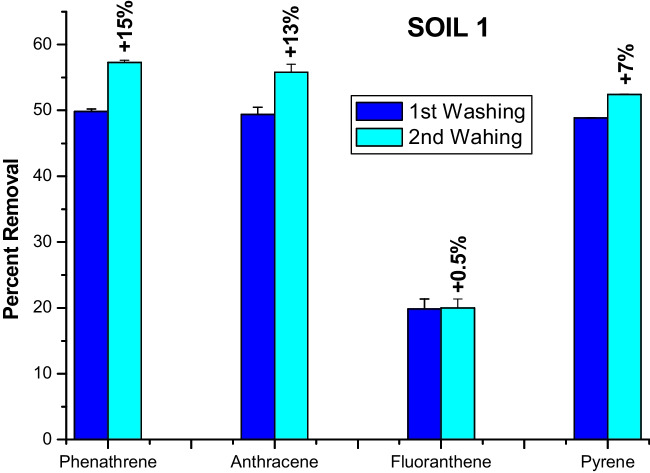
Percent removal of four PAHs from soil 1 by first washing and percent increment obtained by a consecutive second washing with alkylated HA at 60% of total acidity

**Fig. 4:**
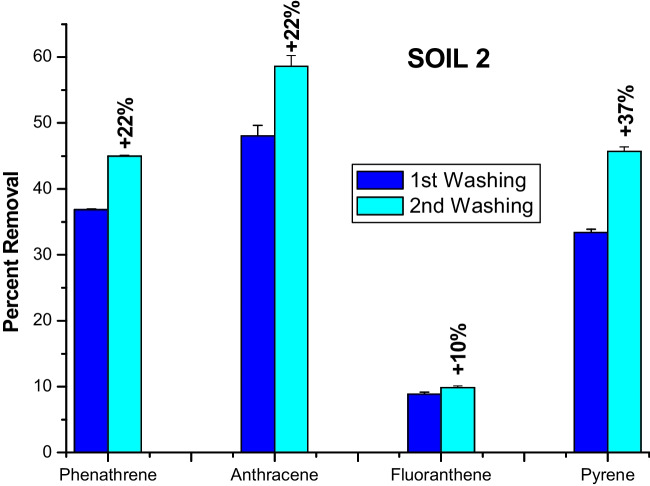
Percent removal of four PAHs from soil 2 by first washing and percent increment obtained by a consecutive second washing with alkylated HA at 60% of total acidity

The efficiency of soil remediation by washing with surfactants is often enhanced by subsequent soil washing cycles (Ishikawa and Oya 2008; Shen et al. 2021). Here, we subjected the soil residue from the first washing with the aqueous solution of the most performing benzylated HA at 60% of total acidity to a second washing with the same modified HA solution and the percent removal of PAHs by the two consecutive washings was calculated (Figs. 3 and 4).

The second washing for soil 1 removed a further amount, in respect to the first washing, of PHE, ANT, and PYR by 15, 13, and 7%, respectively, while it slightly varied the release of FLA just by 0.5% (Fig. 3). In the case of soil 2, the second washing solubilized an additional share of the PHE, ANT, PYR, and FLA content by 22, 22, 10, and 37%, respectively (Fig. 4).

Previous experiments on pollutant removal from soil by consecutive washings had not been successful. Three consecutive cycles of soil washing by SDS failed to remove PHE by any significant improvement with cycles (Wang et al. 2020). Similarly, the consecutive applications of different biosurfactants and synthetic Triton X-100 to wash PHE off a soil showed that a single soil wash was enough to remove most of the phenanthrene (Lima et al. 2011). Contrary to these reports, a humic material empowered by the inclusion of benzyl groups was found here to significantly enhance its surfactant properties and removed further amounts of PAHs in the second soil washing. Such a heightened functionality of the benzylated HA in removing PAHs from soils is to be attributed to both the greater repartition of pollutants into the more abundant hydrophobic domains of this humic derivative and to the increased potential to form π-π bonds between benzyl groups and PAH aromatic rings. Moreover, the greater pollutant removal in the second washing for soil 2 than for soil 1 (Figs. 3 and 4) may be explained by the different textural characteristics of the two soils. In fact, it can be hypothesized that the first washing with the highly hydrophobic benzylated HA may have altered the aggregates arrangement of the finer textured soil 2 more than the coarse soil 1 (Jastrow 1996), thereby favoring the disruption of some macroaggregates into microaggregates (Conte et al. 2005). This process may then have exposed a larger share of microaggregate surfaces to the aqueous solution of the benzylated HA during the second soil washing and enhanced the repartition of adsorbed PAHs from soils into the hydrophobic phases of the modified HA.

## Conclusions

The structural modification of a humic acid with the covalent insertion of methyl, pentyl, and benzyl groups was shown here to increase its surfactant capacity and improve significantly its soil washing efficiency. This finding is attributed to the enhanced HA hydrophobicity that favored PAH repartition from soil particle surfaces into humic apolar domains. Removal of PAHs from a loamy-sandy-clay soil by washing with all O-alkylated derivatives at different rates of HA total acidity showed an invariable increase, with respect to the unmodified HA, that was up to 20-fold when fluoranthene was solvated by benzylated HA. A similar behavior was shown by the finer textured clay-loam soil, though to a lesser extent. For both soils, the best efficiency in soil washing was shown when O-alkylation was at 60% of the humic total acidity and the benzylated HA was generally the most performing among all derivatives. Such greater efficiency in removing PAHs from soils is attributed to the disruption of soil aggregation by the enhanced surfactant capacity of benzylated HA and to the formation of π-π bonds between the pollutants’ aromatic rings and the newly introduced benzyl groups of the benzylated derivative. Alkylated HA showed better PAH removal capacity than the nonionic synthetic surfactant Triton X-100, whereas only the benzylated and methylated HA appeared to nearly and fully match, respectively, the soil washing efficiency of the SDS anionic surfactant. A significant increase in PAH release was observed when soils were washed consecutively by a second 60% benzylated HA solution, since it may have further induced the disruption of soil aggregates and the exposure of a greater surface of soil particle to the washing. Our findings indicate that a simple and low-cost O-alkylation reaction can significantly increase the potential of a natural surfactant, such as a humic acid, in washing PAHs from a contaminated soil by an efficiency comparable to that of synthetic surfactants. While the extent of pollutant removal appears still dependent on soil texture, this limitation may be overcome by efficiently applying benzylated HA solutions in consecutive washings, without the risk of introducing excessive biological toxicity in the environment, as it is the case for synthetic surfactants.

### Supplementary Information

Below is the link to the electronic supplementary material.

Supplementary file1 (DOCX 53 KB)
