# Supplementary material for: Enhanced washing of polycyclic aromatic hydrocarbons from contaminated soils by the empowered surfactant properties of de novo O-alkylated humic matter
Source: Environ Sci Pollut Res Int. 2024 Feb 8;31(11):16995–7004. doi: 10.1007/s11356-024-32292-3 (PMC10894171; doi:10.1007/s11356-024-32292-3)
Supplement: Supplementary file 1 — Supplementary file1 (DOCX 53 KB) [file 11356_2024_32292_MOESM1_ESM.docx]

**SUPPORTING INFORMATION**

**Enhanced washing of Polycyclic Aromatic Hydrocarbons from contaminated soils by the empowered surfactant properties of *de novo* O-alkylated humic matter**

Alessandro Piccolo^1*^, Marios Drosos^2^, Assunta Nuzzo^1^, Vincenza Cozzolino^1^, Antonio Scopa^2^

1. Department of Agricultural Sciences, University of Naples Federico II, Via Università 100, 80055 Portici, Italy.
2. School of of Agricultural, Forestal, Food and Environmental Sciences (SAFE), University of Basilicata, Viale dell’Ateneo Lucano 10, 85100, Potenza, Italy

*. Corresponding Authors: alessandro.[piccolo@unina.it](mailto:piccolo@unina.it);

**Table S1**. Percent increase of PAHs removal from soil 1 and soil 2 by washings with aqueous solutions of alkylated HA at 40, 60, and 80 % of total acidity, as compared to washings by the original unmodified HA.

|  | **Phenanthrene** | **Anthracene** | **Fluoranthene** | **Pyrene** |
| --- | --- | --- | --- | --- |
|  | **SOIL 1** | | | |
|  | **Methylated HA** | | | |
| **40** | 57.9±8.4 | 31.6±1.3 | 1720.0±400.0 | 135.1±11.9 |
| **60** | 63.2±1.2 | 26.7±0.4 | 1660.0±460.0 | 141.7±0.6 |
| **80** | 34.1±10.0 | 26.2±3.3 | 1160.0±580.0 | 111.3±16.7 |
|  | **Pentylated HA** | | | |
| **40** | 70.9±5.0 | 23.7±0.2 | 1680.0±200.0 | 137.5±7.7 |
| **60** | 63.2±0.8 | 24.1±1.6 | 2520.0±60.0 | 117.9±2.3 |
| **80** | 57.9±2.6 | 21.6±0.2 | 860.0±160.0 | 107.7±5.4 |
|  | **Benzylated HA** | | | |
| **40** | 51.0±0.3 | 6.0±0.7 | 1780.0±720.0 | 82.7±9.0 |
| **60** | 90.8±1.5 | 14.6±2.4 | 3880.0±300.0 | 191.1±0.6 |
| **80** | 73.9±5.4 | 5.8±2.3 | 2500.0±300.0 | 137.5±7.2 |
|  | **SOIL 2** | | | |
|  | **Methylated HA** | | | |
| **40** | 118.3±6.7 | 32.9±0.2 | 516.7±66.6 | 202.7±7.2 |
| **60** | 138.3±3.9 | 57.7±1.4 | 1683.3±50.0 | 275.7±3.6 |
| **80** | 136.7±7.7 | 57.1±3.2 | 1616.7±400.0 | 273.0±7.2 |
|  | **Pentylated HA** | | | |
| **40** | 79.4±2.8 | 15.1±8.9 | -66.7±66.7 | 133.3±19.0 |
| **60** | 94.4±6.7 | 17.1±2.0 | 216.7±116.6 | 137.8±0.9 |
| **80** | 76.1±6.7 | 17.4±2.6 | 250.0±250.0 | 128.8±11.7 |
|  | **Benzylated HA** | | | |
| **40** | 70.0±12.8 | 35.4±1.7 | -33.3±33.3 | 95.5±23.4 |
| **60** | 105.0±0.6 | 37.1±4.6 | 1383.3±50.0 | 200.9±4.5 |
| **80** | 70.5±12.3 | 38.6±0.8 | 400.0±400.0 | 145.0±13.6 |

**Fig. S1.** Percent removal of four PAHs from Soil 1 by water, unmodified HA and HA methylated at 40, 60 and 80 % of total HA acidity.

**Fig. S2.** Percent removal of four PAHs from Soil 2 by water, unmodified HA and HA methylated at 40, 60 and 80 % of total HA acidity.

**Fig. S3.** Percent removal of four PAHs from Soil 1 by water, unmodified HA and HA pentylated at 40, 60 and 80 % of total HA acidity.

**Fig. S4.** Percent removal of four PAHs from Soil 2 by water, unmodified HA and HA pentylated at 40, 60 and 80 % of total HA acidity.

**Fig. S5.** Percent removal of four PAHs from Soil 1 by water, unmodified HA and HA benzylated at 40, 60 and 80 % of total HA acidity.

**Fig. S6.** Percent removal of four PAHs from Soil 2 by water, unmodified HA and HA benzylated at 40, 60 and 80 % of total HA acidity.
